# MATURE RELIGION AT 75: AN APPRAISAL OF GORDON ALLPORT’S (1950) “THE INDIVIDUAL AND HIS RELIGION”

**DOI:** 10.1093/geroni/igae098.2553

**Published:** 2024-12-31

**Authors:** Kelly Mirarchi, Peter Hope, Delaney Sommers, Edward Thompson, Michiko Iwasaki, Andrew Futterman

**Affiliations:** Loyola University Maryland, Baltimore, Maryland, United States; Johns Hopkins University School of Medicine, Baltimore, Maryland, United States; Loyola University Maryland, Baltimore, Maryland, United States; College of the Holy Cross, Worcester, Massachusetts, United States; Loyola University Maryland, Baltimore, Maryland, United States; Loyola University Maryland, Baltimore, Maryland, United States

## Abstract

In his classic manuscript, Gordon Allport (1950) presented his theory of the development of the “religious sentiment”. Allport posited that personal religiousness is similar to other “sentiments” (i.e., guiding principles of behavior) and matures for some individuals. For those who are religiously-oriented, religious behavior is enacted in childhood first to obtain diffuse social and emotion benefits. As these individuals age, religious behavior becomes more intrinsically motivated, and religion directs and integrates adult life. The transition from child-like, extrinsically motivated religion to a more mature religion occurs, for Allport, usually in midlife, as religious doubts are resolved in the course of daily life. We examine Allport’s theory of religious maturity in light of recent research on aging and religious development, research on complexity of thought and emotion in later life, and our own study of religious complexity in later life involving 278 open-ended interviews of individuals aged 55-101. We argue that, in many respects, Allport’s theory has stood the test of time – nearly 75 years - remarkably well. For one thing, Allport (1950) anticipates the main finding of recent research on complexity and aging and is in keeping with findings of our own study, that older adults tolerate their doubts (in this case, their religious doubts) better as they age, and test, affirm, and sometimes change beliefs through social interactions. From our findings, for many individuals, their personal religiousness does seem become more mature – more intrinsic, more integrated, more directing – just as Allport posited.

